# Characterization of Structure and Function of ZS-9, a K^+^ Selective Ion Trap

**DOI:** 10.1371/journal.pone.0114686

**Published:** 2014-12-22

**Authors:** Fiona Stavros, Alex Yang, Alejandro Leon, Mark Nuttall, Henrik S. Rasmussen

**Affiliations:** 1 ZS Pharma Inc., Coppell, Texas, United States of America; 2 Xelay Acumen, Inc., Belmont, California, United States of America; RMIT University, Australia

## Abstract

Hyperkalemia, a condition in which serum potassium ions (K^+^) exceed 5.0 mmol/L, is a common electrolyte disorder associated with substantial morbidity. Current methods of managing hyperkalemia, including organic polymer resins such as sodium polystyrene sulfonate (SPS), are poorly tolerated and/or not effective. Sodium zirconium cyclosilicate (ZS-9) is under clinical development as an orally administered, non-absorbed, novel, inorganic microporous zirconium silicate compound that selectively removes excess K^+^ *in vivo*. The development, structure and ion exchange properties of ZS-9 and its hypothesized mechanism of action are described. Based on calculation of the interatomic distances between the atoms forming the ZS-9 micropores, the size of the pore opening was determined to be ∼3 Å (∼diameter of unhydrated K^+^). Unlike nonspecific organic polymer resins like SPS, the ZS-9 K^+^ exchange capacity (KEC) was unaffected by the presence of calcium (Ca^2+^) or magnesium ions (Mg^2+^) and showed>25-fold selectivity for K^+^ over either Ca^2+^ or Mg^2+^. Conversely, the selectivity of SPS for K^+^ was only 0.2–0.3 times its selectivity for Ca^2+^ or Mg^2+^in mixed ionic media. It is hypothesized that the high K^+^ specificity of ZS-9 is attributable to the chemical composition and diameter of the micropores, which possibly act in an analogous manner to the selectivity filter utilized by physiologic K^+^ channels. This hypothesized mechanism of action is supported by the multi-ion exchange studies. The effect of pH on the KEC of ZS-9 was tested in different media buffered to mimic different portions of the human gastrointestinal tract. Rapid K^+^ uptake was observed within 5 minutes - mainly in the simulated small intestinal and large intestinal fluids, an effect that was sustained for up to 1 hour. If approved, ZS-9 will represent a novel, first-in-class therapy for hyperkalemia with improved capacity, selectivity, and speed for entrapping K^+^ when compared to currently available options.

## Introduction

Hyperkalemia, a condition in which serum potassium ions (K^+^) exceed 5.0 mmol/L [1], is a common electrolyte disorder present in up to 10% of hospitalized patients [2]. Because K^+^ is primarily excreted by the kidneys [3], hyperkalemia often occurs in patients with impaired renal function. The risk of developing hyperkalemia is increased by the use of drugs that disrupt K^+^ balance such as K^+^ sparing diuretics and blockers of the renin-angiotensin-aldosterone system (RAAS) (e.g., angiotensin-converting enzyme [ACE] inhibitors, angiotensin receptor blockers, spironolactone) [2], [4]–[6]. Although hyperkalemia is generally asymptomatic, it impairs cardiac function, may lead to life-threatening arrhythmias [7] and is an independent risk factor for cardiovascular mortality [8].

Three primary management strategies are used to treat patients with chronic hyperkalemia; removing or replacing drugs that cause hyperkalemia [e.g., K^+^ sparing diuretics, RAAS inhibitors], improving compliance with dietary K^+^ restriction; and increasing K^+^ excretion [9]. However, none of these approaches are optimal. A K^+^ restricted diet is difficult for many patients to maintain and is often insufficient to fully control hyperkalemia. Although teatment with spironolactone, RAAS and ACE inhibitors has been shown to provide cardiovascular benefit and reduce mortality in patients with heart failure [10]–[12], these drugs also have an increased risk of hyperkalemia. The only available United States Food and Drug Administration (FDA)–approved product to treat hyperkalemia is the nonspecific organic ion exchange polymer resin, sodium polystyrene sulfonate (SPS) [13]. However, SPS is poorly tolerated, has low selectivity for K^+^, cannot be used in salt-sensitive subjects and has a tendency to cause severe constipation [14]. Although SPS is currently available and widely used in suspension with the laxative sorbitol, cases of serious gastrointestinal adverse events and colonic necrosis were reported with this combination, culminating in a 2009 FDA recommendation that SPS should not be used in combination with sorbitol [15]. Moreover, questions about efficacy have also been raised. In a prospective study in healthy subjects, the addition of SPS to laxative regimens resulted in only marginal increases in K^+^ excretion compared to laxatives alone [16]. A safer and more effective alternative to organic polymer resins, like SPS, is clearly needed to treat hyperkalemia.

### Development of sodium zirconium cyclosilicate (ZS-9)

Zirconium (Zr), which is present throughout the environment [17], has been used extensively in both dental and medical applications because of its biocompatibility and very low toxicity for the past 40+ years. Zirconium silicates were initially identified to selectively extract ammonium from cation mixtures [18], [19]. Investigations of the clinical use of zirconium silicates included removing cations (i.e., ammonium) from dialysis filters [20]. Ammonium and K^+^ cations have similar diameters, suggesting that zirconium silicates might be effective in capturing K^+^. Thus, an optimization process was undertaken to identify a microporous zirconium silicate drug with an optimal balance of counter ions and a pore opening that would selectively capture K^+^ cations. This was accomplished through the screening of an 11-member microporous zirconium silicate family of compounds and by altering the counter ions present in the drug candidates. This process resulted in selection of ZS-9, which mimicked the action of physiologic K^+^ channels.

**Figure 1:**
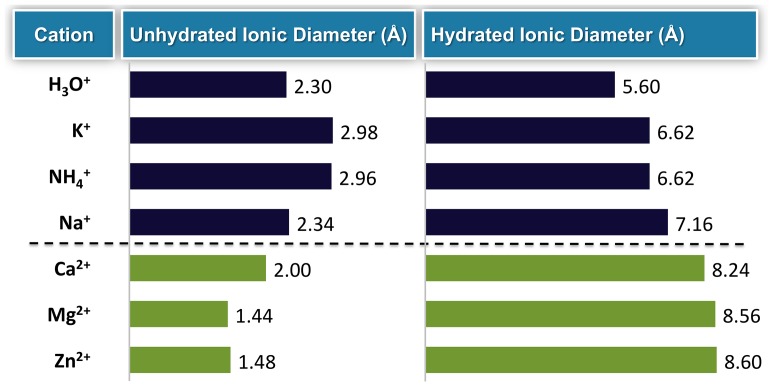
Diameters (Å) of monovalent and divalent cations (both unhydrated and hydrated). Published radiuses of hydrated ions are statistical averages of a population of ions [35].

**Figure 2:**
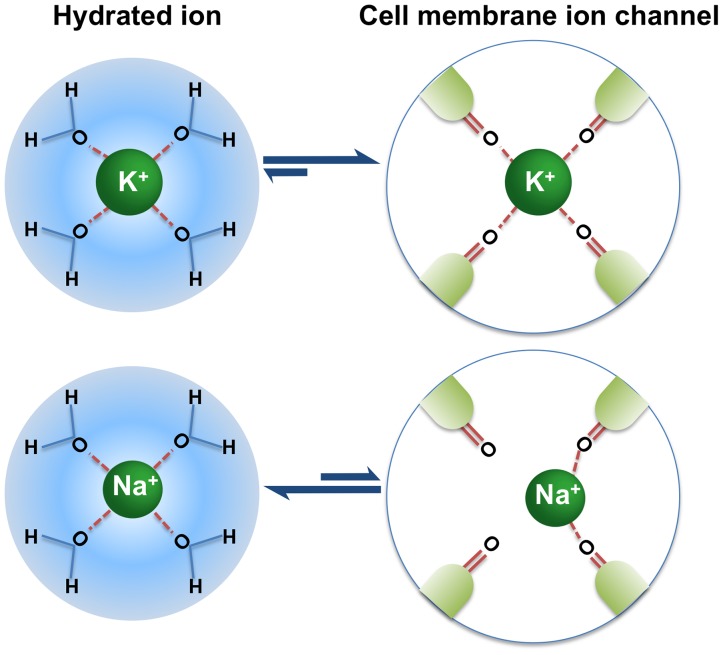
K^+^ specificity of the selectivity filter of a physiologic K^+^ channel. After shedding its coat of water, interaction with the oxygens makes it energetically favorable for K^+^ ions to pass through the channel; however, this action is energetically unfavorable for Na^+^ ions, as they are too small to interact with the oxygens. Adapted from Fulton, TB [22].

Physiologic ion channels take advantage of the differing diameters of ions to selectively filter certain cations (Fig. 1). For example, although K^+^ and sodium ions (Na^+^) are similar in ionic size, K^+^ membrane channels discriminate between K^+^ and Na^+^ by a factor of 10,000 [21]. This is accomplished by the use of a selectivity filter (Fig. 2) [22]. In order to pass through this selectivity filter, an ion must first shed its hydration sphere so as to interact with the carbonyl oxygens at the pore entrance. Although the selectivity filter is large enough to accommodate both K^+^ and Na^+^, only K^+^ are of a sufficient size to interact effectively with the ion channel carbonyl oxygens after shedding their hydration shells. Since the K^+^–oxygen interactions balance the energy lost due to the removal of the water molecules (i.e., carbonyl oxygen in the ion channel mimic the hydration shell), it becomes energetically favorable for K^+^ to move through the filter. However, when Na^+^ shed their hydration shells, they are too small to interact effectively with the optimal number of oxygen molecules in the ion channels, making it energetically unfavorable for them to pass through the filter. Due to the pore size and composition, microporous zirconium silicate drug candidates can effectively mimic the highly selective nature of physiologic K^+^ channels in the body.

During the drug development process, it was important to select counter ions that would be appropriate for the target patient population. Aside from limited ability to take up Ca^2+^, there are a number of clinically relevant reasons why a Ca^2+^ counter ion would be a poor candidate. The target population for ZS-9 therapy is likely to be patients with chronic kidney disease (CKD), who are at increased risk for hyperkalemia as the kidney is responsible for>90% of K^+^ excretion. Since CKD patients have difficulty buffering increased Ca^2+^ loads, the National Kidney Foundation Kidney Disease Outcomes Quality Initiative guidelines caution against high Ca^2+^ intake in these patients [23]. Because SPS uses 100% Na^+^ as the counter cation in the cation exchange, there have been issues with Na^+^ loading due to the lack of cation specificity that necessitates the use of high doses of SPS to achieve efficacy [14]. For this reason, 100% Na^+^ was also not considered ideal. Ultimately it was decided that partial protonation (i.e., Na^+^–H^+^) would provide the optimum composition.

The form of ZS-9 under development for therapeutic use is a non-absorbed, insoluble, free-flowing, odorless, tasteless, white crystalline powder with a specific particle size distribution profile>3 µm. The molecular formula of sodium zirconium cyclosilicate has a specific, and constant, ratio of zirconium and silicon atoms and contains less than ∼8% Na^+^ by total weight. Unlike organic polymers such as SPS, ZS-9 is an inorganic cation exchange crystalline compound that has a high capacity to selectively entrap monovalent cations, specifically excess K^+^ and ammonium ions, over divalent cations such as Ca^2+^ and magnesium (Mg^2+^), as it traverses the GI tract. Because ZS-9 is not systemically absorbed, the risk of systemic toxicity is very low, as demonstrated in three prospective, randomized, double-blind, placebo controlled studies in over 1100 patients [24]–[26]. Additionally, unlike SPS, ZS-9 does not absorb water (i.e., swell) within the GI tract and therefore is not expected to be associated with the GI side effects associated with nonspecific organic polymers like SPS.

Based on the high specificity of ZS-9 to entrap K^+^, the aim of this publication was to characterize the structure, mechanism of action, and ion exchange properties of ZS-9 as a potential therapeutic in the treatment of patients with hyperkalemia.

## Materials and Methods

### Calculation of the size of the ZS-9 pore opening

The interatomic distances of atoms forming the pore of ZS-9 were calculated from the Rietveld structural refinement. For this, a sample of ZS-9 was dried and ground in an agate mortar, then placed into a powder X-ray diffractometer. Data were collected at room temperature from 5–80° 2-theta with monochromated Cu α_1_ radiation (λ = 1.5406 Å) using a MiniFlex-600 diffractometer (Rigaku Corp., Tokyo, Japan). The software suite Jade 9.5 Professional (Materials Data, Inc., Livermore, CA) was used to perform the full pattern least squares Rietveld calculations. With the refined atom positions, the interatomic distances were calculated using ATOMS version 6.0 (Shape Software, Kingsport, TN). The size of the pore opening was calculated by subtracting twice the atomic radius of oxygen (van der Waals radius, r = 1.52 Å) from center–center oxygen–oxygen interatomic distances.

### Ion exchange capacities of ZS-9 and SPS

Equal concentrations (100–200 mg) of ZS-9 or SPS was added to 20 mL solutions containing standard ppm concentrations of K^+^, in addition to Ca^2+^ and Mg^2+^ at different ratios (1∶0.5∶0.5, 1∶1∶1, 1∶2∶2). After 2 hours of gentle agitation at room temperature, an aliquot was taken from the supernatant and the cations quantified by ion chromatography using a Dionex Model ICS-1100 (Thermo Scientific, USA), coupled to an AS-DV autosampler.

### The effect of pH on the K^+^ exchange capacity of ZS-9

The effect of pH on the KEC of ZS-9 at 0.5, 5 or 50 mg/ml was determined in media buffered to mimic the pH of different portions of the human gastrointestinal (GI) tract and which contained a specified amount of K^+^ [27]. The media tested consisted of water (pH 6.1), simulated gastric fluid minus enzymes (SGF, <pH∼1.2), simulated intestinal fluid (pH 6.8) and buffer (pH 5.5). Samples were incubated for three hours, at ambient temperature, with constant agitation after which the potassium concentration was determined by ion chromatography (IC) using a Dionex ICS 3000 (Thermo Scientific, USA).

In a second study, ZS-9 (2 mg/ml) was incubated in solutions containing 2500–3500 ppm K^+^ at pH 1.2 (SGF), pH 4.5 (simulated small intestinal fluid; SSIF) or at pH 6.8 (simulated large intestinal fluid, SLIF) at 37°C with constant agitation. Aliquots of 5 mL of the solutions were removed at 0, 5, 10, 20, 30 min and 1, 2, 4, 8 and 24 hrs and the K^+^ concentration quantified by atomic emission spectroscopy using a Shimadzu AA6200 spectrophotometer (Shimadzu Corporation, Kyoto, Japan) and an air–acetylene gas mixture.

## Results

### Structure of ZS-9

**Figure 3:**
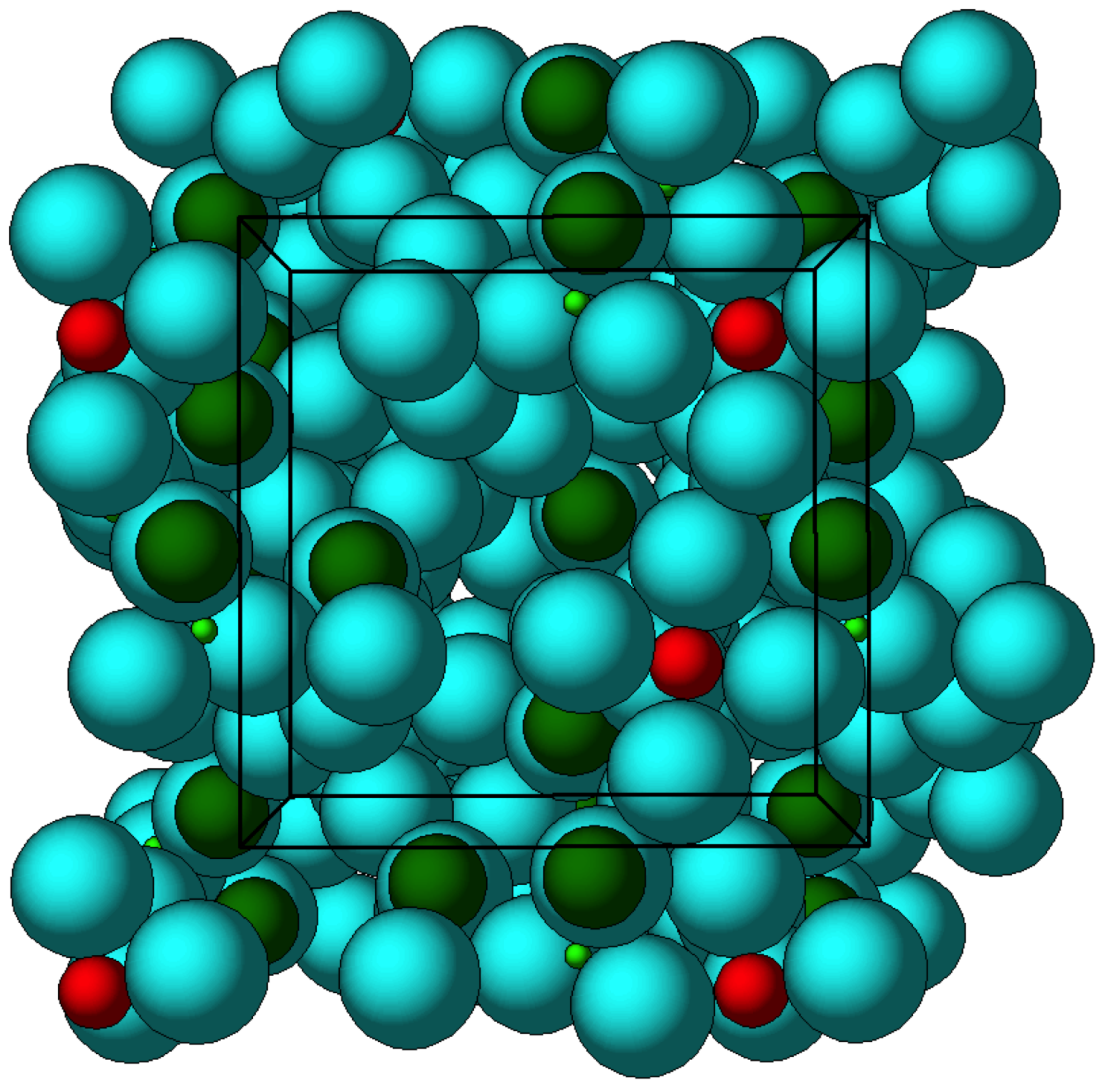
Structure of ZS-9. Blue spheres  =  oxygen atoms; red spheres  =  zirconium atoms; green spheres  =  silicon atoms.

Based on the structural solution for zirconium silicate proposed by Bem et al. [28], a new set of atom positions were generated and refined to convergence and residual statistics of 7.6%. The ZS-9 structure is composed of octahedrally and tetrahedrally oxygen-coordinated Zr and Si atoms, respectively. Oxygen atoms are shared between these complexes and act like bridges between units to form a well-ordered microporous cubic structure (Fig. 3). The Zr–O and Si–O bonds are mainly covalent in nature and the lattice framework acquires its negative charge due to the octahedral [ZrO_6_]^-2^ units. The counter ions located within the micropore channels provide electrical neutrality to the structure. The framework–counter ion interactions are electrostatic in nature, i.e., ionic bonds.

**Figure 4:**
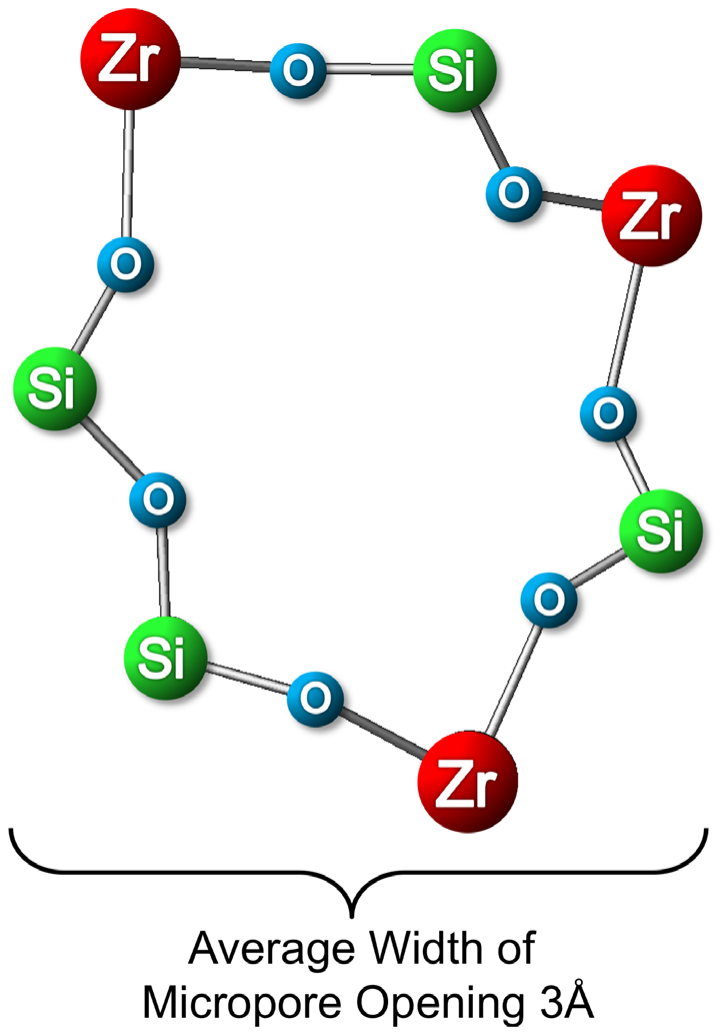
Pore opening of ZS-9 (stick and ball representation).

Detailed analysis of the structure reveals that the pore opening in the ZS-9 framework is composed of an asymmetrical seven-member ring (oxygen atoms are not considered in the count, [Fig. 4]). On average the size of the ZS-9 pore opening was estimated to be ∼3 Å.

### ZS-9 ion exchange characterization

The exchange capacities of ZS-9 and SPS for the different solutions containing K^+^, Ca^2+^ and Mg^2+^ are summarized in Table 1. The exchange capacities of ZS-9 for the divalent ions Ca^2+^ and Mg^2+^ were below 0.05 mEq/g, the analytical method lower limit of quantification (LLOQ). Based on this LLOQ, ZS-9 had a>25-fold selectivity for K^+^ over either Ca^2+^ or Mg^2+^, whereas the selectivity of SPS for K^+^ was only 0.2–0.3 times its selectivity for Ca^2+^ or Mg^2+^. SPS was found to be highly selective for Ca^2+^.

**Table 1:** The ion exchange capacities of ZS-9 and SPS in solutions containing different concentrations of K^+^, Ca^2+^, and Mg^2+^.

Agent	Ratio (K^+^:Ca^2+^:Mg^2+^)	Exchange capacity for K^+^ (mEq/g)	Exchange capacity for Ca^2+^ (mEq/g)	Exchange capacity for Mg^2+^ (mEq/g)
ZS-9	1∶0.5∶0.5	2.8	<LOD^a^	<LOD^a^
ZS-9	1∶1∶1	2.7	<LOD^a^	<LOD^a^
ZS-9	1∶2∶2	2.6	<LOD^a^	<LOD^a^
SPS	1∶0.5∶0.5	0.5	1.0	0.5
SPS	1∶1∶1	0.3	1.0	0.4
SPS	1∶2∶2	0.3	1.1	0.4

Abbreviations: LOD, limit of detection; SPS, sodium polystyrene sulfonante.

a LLOQ (see text) <0.05 mEq/g.

### Effect of pH on ZS-9 function

The ZS-9 KEC increased proportionally with concentration over the 0.5 to 50 mg/mL ZS-9 range tested. There was no significant effect of pH on the KEC of ZS-9 at 50 mg/mL but at ≤5 mg/mL there is a dose dependent decrease in KEC with decreasing pH. At a pH of approximately 1.2 and at concentrations of 0.5 and 5.0 mg/mL the binding capacity of ZS-9 was decreased by 91 and 67%, respectively compared to the binding capacity observed in neutral media (Table 2).

**Table 2:** Effect of pH on KEC at different pH: % Change in KEC vs Control (water).

ZS-9 Concentration mg/ml	% Control KEC		
	SGF pH 1.2*	Buffer pH 5.5*	SIF pH 6.8*
0.5	9	79	87
5.0	33.3	99	102
50.0	91	91	100

*pH of starting solution.

The concentrations of K^+^ over time in the different pH media following incubation with ZS-9 in the second study are presented in Table 3. In the simulated gastric fluid (pH∼1.2), there was an initial small drop in the concentration of K^+^, which reversed soon thereafter. In the simulated small intestinal fluid (pH∼4.5), there was an immediate uptake of K^+^, followed by a small release, with equilibrium reached in about 20 minutes. In the simulated large intestinal fluid (pH 6.8), there was a rapid uptake of K^+^ during the first 10 minutes, followed by continued, but slower, uptake (Table 3) over the next hour with no further increase thereafter. These results show that in environments mimicking the human GI tract and in the presence of ZS-9, K^+^ equilibrium was reached relatively quickly (i.e., in less than 20 minutes).

**Table 3:** Change in K^+^ concentrations over time in solutions containing ZS-9 at different pH.

	K^+^ concentration (ppm) at different time points					
	0 min	5 min	10 min	20 min	30 min	60 min^a^
Simulated gastric fluid (pH 1.2)	112	107	110	111	110	115
Simulated small intestinal fluid (pH 4.5)	101	80	88	93	90	89
Simulated large intestinal fluid (pH 6.8)	139	130	124	126	123	121

a No further change from 1–24 hours.

## Discussion/Conclusions

The substantial differences in the sizes of monovalent and divalent cations (Fig. 1) can be used for targeted drug design. The diameter of the free, unhydrated K^+^ ion is similar to that of the ZS-9 pore (∼3.0 Å), indicating that K^+^ cations can fit more effectively within the lattice structure of ZS-9 after they shed their hydration shell.

**Figure 5:**
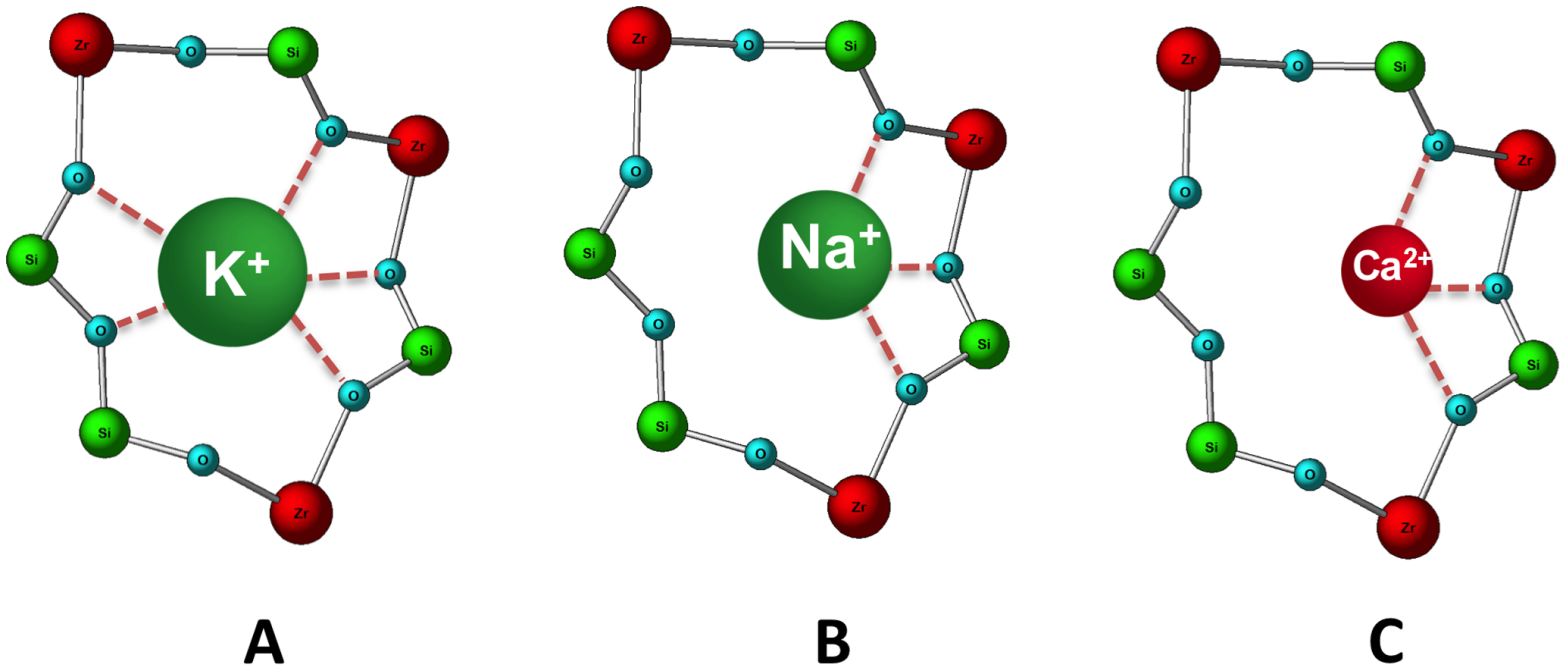
ZS-9 pore detail with a potassium ion (A), a sodium ion (B), and a calcium ion (C).

It is hypothesized that ZS-9 uses a selectivity filter analogous to that of physiologic K^+^ channels to achieve its selectivity for capturing K^+^ ions. Hydrated cations, such as Na^+^ and Ca^2+^, have larger ionic diameters (Fig. 1) and require more energy to shed their hydration shell than K^+^. As shown by the pore opening size data, unhydrated Na^+^ and Ca^2+^ ions (Fig. 1) are too small to maximize the interactions with the oxygen atoms in the ZS-9 pore opening (Figs. 5A and 5B, respectively), making it energetically unfavorable for them to enter. In contrast, the energy to dehydrate the K^+^ is more than balanced by the energy regained by the interaction with the oxygen atoms within the ZS-9 crystal lattice (Fig. 5C).

The selectivity of ZS-9 for K^+^ is confirmed by the ion exchange characterization data, which showed that in mixed cation solutions, ZS-9 had 9.3 times more capacity for K^+^ than SPS and, at all concentration ratios tested, it was more than 125 times more selective for K^+^ compared with SPS. Results from a thermodynamic study have also shown that the exchange of Na^+^ for K^+^ in ZS-9 is more stable by 20 kcal/mol [29]. The ion exchange data also demonstrated that SPS has higher selectivity for Ca^2+^ than for K^+^, which confirms the results reported by Berlyne and colleagues [30] that SPS showed the affinity trend Ca^2+^>Mg^2+^>K^+^ (∼NH_4_^+^)>Na^+^>H^+^. For non specific compounds the majority of the potassium exchange is confined to the colon where there is a large K+/Na+ ratio (8∶1) but due to its high specificity for potassium, ZS-9 is expected to work along the entire length of the GI tract.

Experiments in environments mimicking the GI tract showed what appears as an early uptake of K^+^ ions by ZS-9 at pH 1.2, which reverses with time. However, as the pH increases, there was a rapid and sustained uptake of K^+^. The pH of the human stomach lumen varies between 1 and 3 [31] and contains protons in addition to sodium, potassium and chloride ions as well as enzymes. The degree of K^+^ uptake is dependent on the amount of ZS-9 present, the composition of the ionic environment and time of contact. While K^+^ uptake is likely limited at very low pH due to a large excess of protons, based on the *in vitro* data, some K^+^ exchange will likely begin in lower pH environments in the presence of higher concentrations of ZS-9 and increase as it passes along the GI tract due to the higher potassium concentrations and luminal pH. This is supported by the fact that statistically and clinically significant decreases in serum K^+^ occurred within 1 hour of dosing in three clinical studies [24]. Results from one Phase 2 (NCT01493024) and two Phase 3 studies (NCT01737697 and NTC02088073 [HARMONIZE]) showed statistically significant reductions in mean serum K^+^ from baseline to one hour after the first 10 g ZS-9 dose compared to respective placebo groups (p = 0.044, p = 0.009, p<0.001, respectively) [24]–[26]. -. Data from both Phase 3 studies of ZS-9 in patients with hyperkalemia (with and without CKD) confirmed the dose dependent rapid onset of action and, as expected based on the mode of action, the greatest reductions in serum K^+^ were observed in patients with the highest baseline serum K^+^ values [24], [26]. Furthermore, in all clinical studies, ZS-9 normalization of serum K^+^ levels was rapidly achieved (median of 2.2 hours) and maintained. ZS-9 exhibited an overall safety profile, including GI tolerability, similar to placebo in all three studies [24]–[26], corroborating the historically low incidence of adverse events with the use of zirconium in clinical settings.

Zirconium has been used extensively in both dental and medical applications because of its high biocompatibility and very low toxicity. Zirconium is widely used in dental implants, middle ear implants, and other restorative practices with large amounts embedded in the body [32]. It also has an equally long history of use in patients with CKD, having been used in hemodialysis, peritoneal dialysis, and hemofiltration, where it comes in direct contact with the bloodstream. There have been over 2 million dialysis treatments with REDY and Sorb columns since 1970 [32], [33] and Fresenius' DIALSORB is currently undergoing review by the FDA. Fresenius is also developing a Zr-based Wearable Artificial Kidney [34]. Daily GI exposure to Zr in the diet has been estimated to be>4.0 mg/day (on average ∼3.5 mg from food and ∼0.65 mg from drinking water) [17]. The amount of soluble Zr released from a 10 g dose of ZS-9 as it traverses pH environments similar to those occurring in the GI tract is approximately less than 0.3 micrograms (unpublished data on file at ZS Pharma, Inc.), indicating that daily dietary exposure to Zr is>5 orders of magnitude higher than that from a 10 g dose of ZS-9.

If approved, ZS-9 will represent a novel, first-in-class therapy for hyperkalemia with improved capacity, selectivity, and speed for entrapping K^+^ when compared to currently available options.
